# Protocol for the derivation and alveolar type 2 differentiation of late-stage lung tip progenitors from the developing human lungs

**DOI:** 10.1016/j.xpro.2024.103201

**Published:** 2024-07-18

**Authors:** Kyungtae Lim, Emma L. Rawlins

**Affiliations:** 1 Wellcome Trust/CRUK Gurdon Institute, University of Cambridge, Cambridge CB2 1QN, UK; 2 Department of Physiology, Development and Neuroscience, University of Cambridge, Cambridge CB2 3DY, UK; 3 Wellcome Trust/MRC Stem Cell Institute, Jeffrey Cheah Biomedical Centre Cambridge Biomedical Campus, Puddicombe Way, Cambridge CB2 0AW, UK; 4 Division of Life Sciences, College of Life Sciences and Biotechnology, Korea University, Seoul 02841, South Korea

**Keywords:** Cell culture, Cell Differentiation, Cell isolation, Cell separation/fractionation, Developmental biology, Flow Cytometry, Organoids

## Abstract

Molecular and cellular mechanisms of human lung alveolar development are poorly understood due to a lack of *in vitro* model systems. This protocol details the isolation, derivation, and genetic modification of lung tip epithelial progenitors from human fetal lungs. It includes steps for isolating distal lung epithelial cells, expanding tip progenitor organoids, culturing tip organoids *in vitro*, and differentiating them into alveolar type 2 cells. This will aid in understanding alveolar differentiation mechanisms and neonatal diseases.

For complete details on the use and execution of this protocol, please refer to Lim et al.^1^

## Highlights

• This protocol describes the isolation and derivation of the human distal lung tip epithelium
• Includes steps for expansion and cryopreservation of Lin^POS^ tip epithelial organoids
• The lung tip organoids can be genetically manipulated
• Differentiation of tip organoids to alveolar type 2 fate can be induced within 7 days

## Abstract (editor highlights)

Publisher’s note: Undertaking any experimental protocol requires adherence to local institutional guidelines for laboratory safety and ethics.

## Graphical abstract

**Figure:**
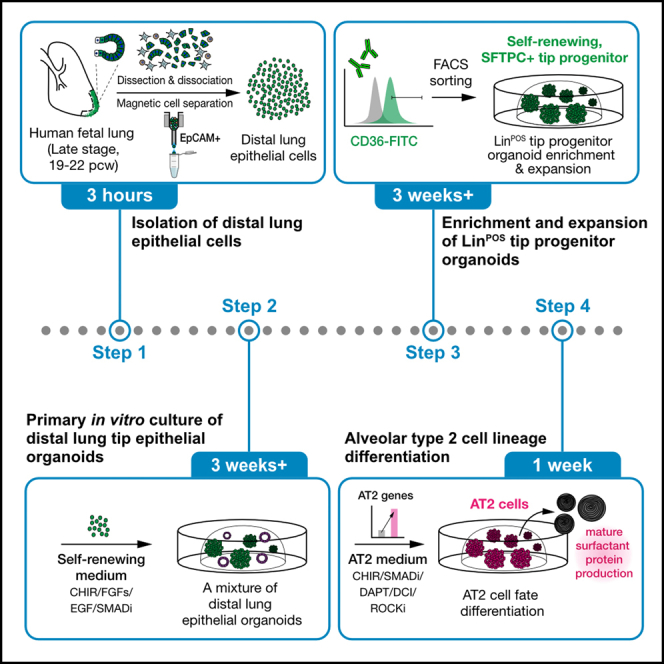

## Before you begin

Multipotent epithelial progenitor cells are found in the distal tip region of the developing lungs.^2^^,^^3^^,^^4^^,^^5^ In the developing human lungs from ∼16 pcw (post-conception weeks) tip epithelial progenitor cells express Alveolar Type 2 (AT2) epithelial cell markers, e.g., SFTPC, and develop into Alveolar Type 1 (AT1) and 2 cells afterwards.^1^^,^^6^ The current protocol describes a method to isolate the distal tip progenitor cells from late-stage (∼19–22 pcw) human fetal lungs and to establish self-renewing, expandable tip progenitor organoid lines that stably express alveolar lineage-associated genes. The tip organoids can be sub-cultured over 20 passages and successfully frozen and thawed as needed. They can also grow from single cells in the presence of a Rho associated Kinase (ROCK) inhibitor, Y-27632, enabling genetic modifications using CRISPR/Cas or lentiviral infection.^1^ Of note, AT2 cell differentiation can be easily achieved *in vitro* within 7 days from the tip progenitor state by simply switching the culture medium. This protocol allows a researcher to readily-derive alveolar-fated, self-renewing fetal lung organoids and use this model system to investigate the molecular mechanisms of human alveolar development and differentiation. Furthermore, the organoids can be also useful for modeling lung diseases related to pulmonary surfactant secretion and trafficking.

**Figure 1:**
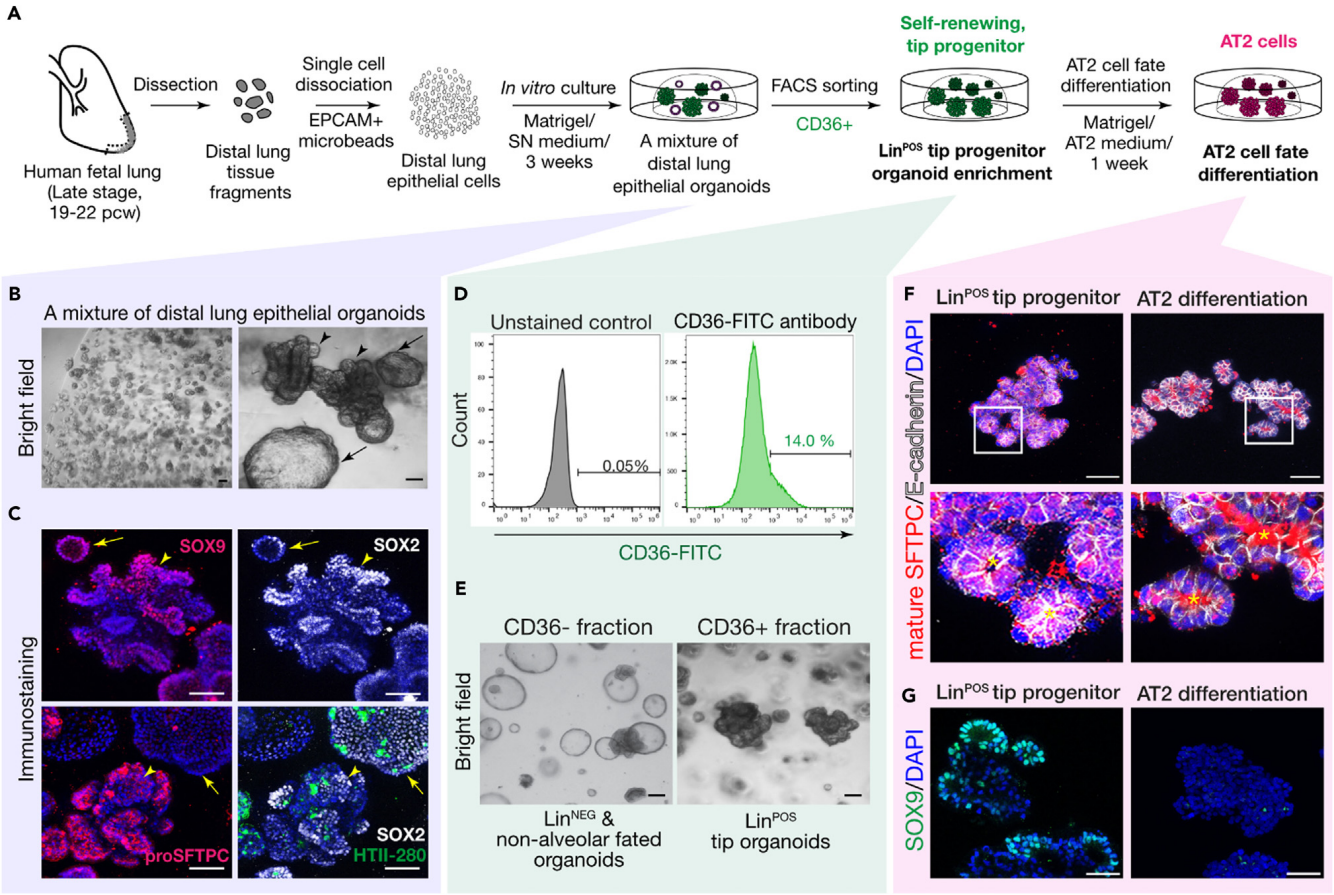
Derivation and *in vitro* organoid culture of late-stage distal lung tip progenitors from human fetal lungs, at 19–21 pcw, and alveolar type 2 (AT2) cell fate differentiation (A) Experimental scheme describing the establishment of alveolar lineage-positive (Lin^POS^) distal tip progenitor organoids and their AT2 cell fate differentiation by exposure to AT2 medium for 1 week. (B and C) Bright field (top panel; B) and immunostaining images (middle and low panels; C) of a mixture of distal lung tip epithelial organoids. Arrowheads and arrows indicate Lin^POS^ (SOX9^+^, SOX2^+^, SFTPC^+^; folded) and lineage-negative (Lin^NEG^) tip organoids (SOX9^+^, SOX2^+^, SFTPC^-^; cystic), respectively. (D) FACS-isolating CD36 positive cells from a mixture of distal lung tip epithelial organoids. Only CD36+ cell subpopulation was purified following single, live-cell gating. (E) Enrichment of alveolar fated, Lin^POS^ tip organoid (right) is achieved by growing the CD36+ fraction, while CD36- fraction gives rise to Lin^NEG^ and non-alveolar organoids (left). (F and G) Immunostaining images comparing self-renewing, alveolar-fated Lin^POS^ tip organoids (left panels) and AT2 organoids (right panels). Scale bars, 50 μm.

This protocol below includes 1) isolating the tip epithelial progenitor cells from human fetal lung tissues, 2) 3D-based *in vitro* culture of the isolated tip progenitor cells for 3 weeks+ (passage 0), 3) enriching the alveolar lineage positive (Lin^POS^) tip organoids, and 4) AT2 cell differentiation and maturation within 7 days (Figure 1). The researchers will need to scale up the cell numbers for next-generation sequencing (NGS), gene editing, or transcription/protein assays that generally require a large number of cells as an input. Optional protocols for lentiviral transduction, continued passaging, and cryopreservation are also included. All the steps below must be carried out in sterile conditions and should be locally risk assessed to comply with region-specific biological safety guidelines.

### Institutional permissions

Human embryonic and fetal lung tissues were provided from terminations of pregnancy from Cambridge University Hospitals NHS Foundation Trust under permission from NHS Research Ethical Committee (96/085) and the MRC/Wellcome Trust Human Developmental Biology Resource (London and Newcastle, University College London (UCL) site REC ref. 18/LO/0822; Newcastle site REC ref. 18/NE/0290; Project 200454; www.hdbr.org). All research projects using human fetal-derived cells should undergo local ethical approval and conform to all the relevant regulatory standards.

### Keep human fetal lungs on ice

1. Place and keep the fetal human lungs on ice while dissolving tissue dissociation buffer.

**Figure 2:**
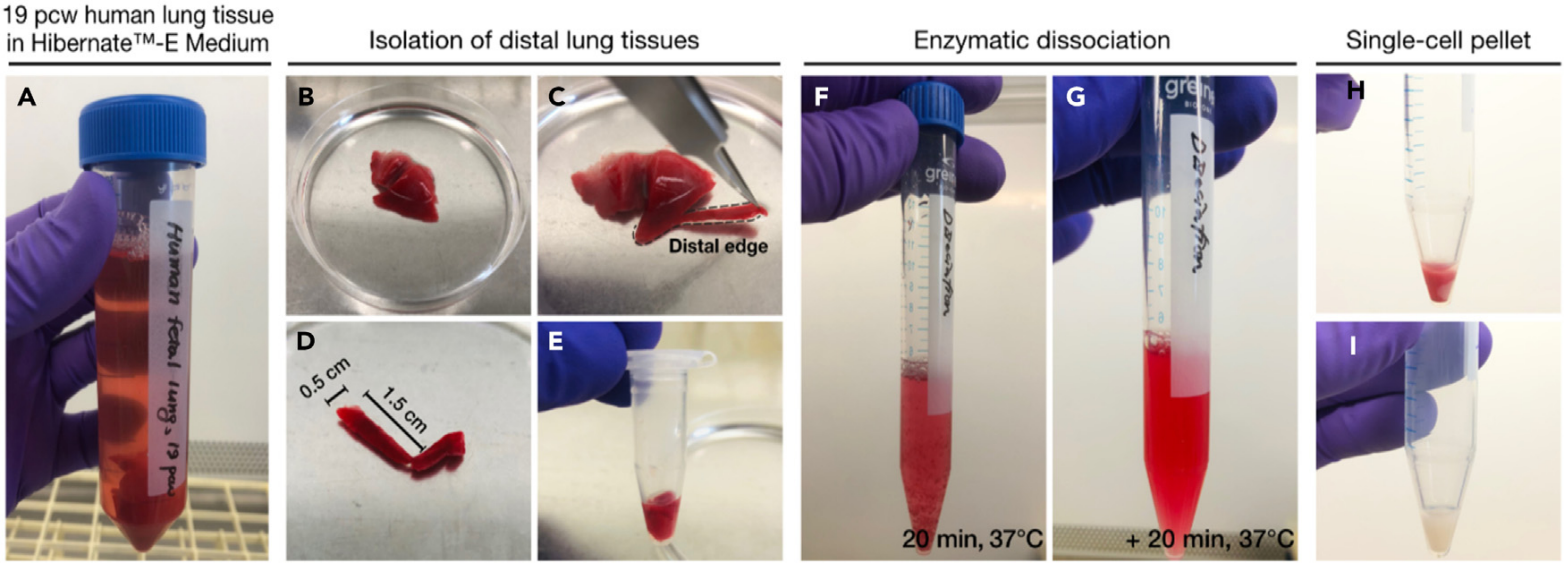
Isolation of distal lung epithelial cells from human lung tissues, steps 1–14 (A) Human lung tissues at 19 pcw kept in cold Hibernate-E medium. (B–E) Isolation of the edge of the lung tissues, followed by mincing into small pieces. (F and G) Enzymatic dissociation of the fragmented pieces into single cells. (H and I) The single-cell pellet before (H) and after (I) removal of red blood cells.

**CRITICAL:** If possible, use tissue immediately on the day of collection. If required, the tissues can be kept in Hibernate-E Medium on ice, up to 24 h (Figure 2A).

### Dissolve tissue dissociation buffer

**Timing: 20 min**

2. Thaw 5 mL tissue dissociation buffer on ice.

### Prepare wash buffer

**Timing: 3 min**

3. Prepare 50 mL tissue washing buffer and keep on ice.

### Prepare self-renewing (SN) medium

**Timing: 15 min**

4. Prepare Advanced DMEM/F12 medium supplemented with 1× GlutaMAX, 1 mM HEPES, and 1× Penicillin/Streptomycin (hereafter referred to as basal medium)
5. Pre-thaw the necessary amount of each supplement, including growth factors and small molecules on ice. Please see the components of SN medium in materials and equipment section.
6. Prepare 40 mL SN medium by adding the supplements to the basal medium.
7. Keep the SN medium in the fridge and use it for up to 1 week.
8. Pre-warm the medium at 37°C water bath for 20 min before use.

### Prepare alveolar type 2 cell (AT2) differentiation medium

**Timing: 15 min**

9. Pre-thaw supplements, including growth factors and small molecules on ice. Please see the detailed components of alveolar differentiation medium (AT2 medium) in materials and equipment section.
10. Prepare 40 mL AT2 differentiation medium by adding the supplements to the basal medium.
11. Keep the medium in the fridge and use for up to 1 week.

**CRITICAL:** Prepare this medium before use and can be stored at 4°C for up to 1 week.

## Key resources table

**Table:** 

REAGENT or RESOURCE	SOURCE	IDENTIFIER
**Antibodies**		

Mouse monoclonal anti-CD36, FITC conjugated (1:100 dilution for use)	Thermo Fisher Scientific	Cat# 11-0369-42, RRID:AB_10718972

**Recombinant DNA**		

Modified pHAGE-hSFTPC-eGFP-W-EF1a-TagRFP	A gift from Darrell Kotton	Addgene plasmid # 36450; http://n2t.net/addgene:36450; RRID: Addgene_36450

**Biological samples**		

Human fetal lung samples	MRC/Wellcome Trust Human Developmental Biology Resource (London, University College London [UCL] site REC reference: 18/LO/0822; Project 200454; www.hdbr.org)	HDBR-L 14996, HDBR-L 14556, HDBR-L 14404, HDBR-L 14559

**Chemicals, peptides, and recombinant proteins**		

Advanced DMEM/F12	Thermo Fisher Scientific	12634–028
GlutaMAX supplement	Thermo Fisher Scientific	35050061
HEPES	Thermo Fisher Scientific	15630080
Penicillin/Streptomycin	Merck	P4333
N2 supplement	Thermo Fisher Scientific	17502001
B27 supplement	Thermo Fisher Scientific	12587001
N-Acetylcysteine	Merck	A9165
Recombinant human EGF	PeproTech	AF-100-15
Recombinant human Noggin	PeproTech	120-10C
Recombinant human FGF10	PeproTech	100–26
Recombinant human FGF7	PeproTech	100–19
CHIR99021	Stem Cell Institute, University of Cambridge	-
SB431542	Bio-Techne	1614
cAMP	Merck	B5386
IBMX	Merck	I5879
Y-27632	Merck	688000
Dexamethasone	Merck	D4902
A-83-01	Tocris	2939
DAPT	Merck	D5942
TrypLE Express enzyme (1×), phenol red	Thermo Fisher Scientific	12605028
Matrigel∗, growth factor reduced (GFR) basement membrane matrix, phenol red-free, LDEV-free	Corning	356231
RBC lysis buffer	BioLegend	420301
Collagenase	Merck	C9891
Dispase	STEMCELL Technologies	07913
DNase	Merck	D4527
Recovery Cell culture freezing medium	Thermo Fisher Scientific	12648010
Hibernate-E medium	Thermo Fisher Scientific	A1247601
Fetal bovine serum (FBS)	PAN-Biotech	P30-3031

**Critical commercial assays**		

Human CD326 (EpCAM) MicroBeads	Miltenyi Biotec	130-061-101
FcR blocking reagent	Miltenyi Biotec	130-059-901
LS columns	Miltenyi Biotec	130-042-401

**Deposited data**		

Bulk-RNA seq data (raw and processed)	Lim et al.^1^	GSE178529
scRNA-seq of lung organoids	Lim et al.^1^	E-MTAB-11435

**Software and algorithms**		

ImageJ (version: 2.1.0)	Schneider et al.	https://imagej.nih.gov/ij/; RRID: SCR_003070
FlowJo software (version: 10.0.0)	FlowJo	FlowJo (https://www.flowjo.com/); RRID: SCR_008520

**Other**		

Scissors	Fine Science Tools	14060-09
Forceps	Fine Science Tools	11295-10
Cell strainer	Merck	CLS431752
Hemocytometer	Marienfeld Superior	0640010
Multiwell plate for suspension culture, 12 well	Greiner	665102
Multiwell plate for suspension culture, 24 well	Greiner	662102
Multiwell plate for suspension culture, 48 well	Greiner	677102

∗Note: Please note that the performance of organoid culture remains consistent regardless of the lot or batch of Matrigel (Corning, 356231).

## Materials and equipment

**Table:** Tissue dissociation buffer

Reagent	Stock concentration	Final concentration	Amount
Advanced DMEM/F12		-	35 mL
Collagenase (powder)	-	0.125 mg/mL	6.25 mg
Dispase	5 U/mL	1 U/mL	10 mL
DNase	1000 U/mL	100 U/mL	5 mL
**Total**		**N/A**	**50 mL**

Note: Store 5 mL (x 10 aliquots), at −20°C. Freshly thaw 5 mL aliquot for each experiment.

**Table:** Tissue washing buffer

Reagent	Final concentration	Amount
Phosphate-buffered saline (PBS; 1×, pH 7.4)	1×	49 mL
Fetal bovine serum (FBS)	2%	1 mL
**Total**	**N/A**	**50 mL**

Note: This buffer should be prepared freshly for immediate use.

**Table:** MACS washing buffer

Reagent	Stock concentration	Final concentration	Amount
PBS	1×	-	48.8 mL
EDTA	0.5 M	2 mM	200 μL
BSA (bovine serum albumin; powder)	-	0.5%	-
**Total**		**N/A**	**50 mL**

Note: This buffer should be prepared freshly for immediate use.

**Table:** FACS buffer

Reagent	Stock concentration	Amount
PBS	1×	49.5 mL
Fetal bovine serum (FBS)	1%	500 μL
**Total**		**50 mL**

Note: This buffer should be prepared freshly for immediate use.

**Table:** Basal medium

Reagent	Stock concentration	Final concentration	Amount
Advanced DMEM/F12 (1:1)	1×	-	475 mL
GlutaMAX supplement	100×	1×	5 mL
HEPES	1 M	1 mM	5 mL
Penicillin/Streptomycin	100×	1×	5 mL
**Total**		**N/A**	**500 mL**

Note: This medium should be stored at 4°C for up to 1 month.

**Table:** Self-renewing (SN) medium

Reagent	Stock concentration	Final concentration	Amount
Basal medium	-	-	38.4 mL
N2 supplement	100×	1×	400 μL
B27 supplement	50×	1×	800 μL
N-Acetylcysteine	250 mM	1.25 mM	200 μL
recombinant human EGF	100 μg/mL	50 ng/mL	20 μL
recombinant human Noggin	100 μg/mL	100 ng/mL	40 μL
recombinant human FGF10	100 μg/mL	100 ng/mL	40 μL
recombinant human FGF7	50 μg/mL	100 ng/mL	80 μL
CHIR99021	10 mM	3 μM	12 μL
SB431542	10 mM	10 μM	40 μL
**Total**		**N/A**	**40 mL**

Note: This medium should be stored at 4°C for up to 1 week.

**Table:** AT2 differentiation medium

Reagent	Stock concentration	Final concentration	Amount
Basal medium	-	-	38.4 mL
N2 supplement	100×	1×	400 μL
B27 supplement	50×	1×	800 μL
N-Acetylcysteine	250 mM	1.25 mM	200 μL
CHIR99021	10 mM	3 μM	12 μL
DAPT	50 mM	50 μM	40 μL
Dexamethasone	50 μM	50 nM	40 μL
cAMP	100 mM	100 μM	40 μL
IBMX	100 mM	100 μM	40 μL
A-83-01	10 mM	10 μM	40 μL
**Total**		**N/A**	**40 mL**

Note: Prepare this medium before use and should be stored at 4°C for up to 1 week.

***Alternatives:*** 10 mM SB-431542 can be used as an alternative to 10 mM A-83-01.

## Step-by-step method details

### Isolation of distal lung epithelial cells from human fetal lungs

**Timing: 3 h**

This section describes the procedure for dissection of distal lung tissues from late-stage human fetal lungs. Careful dissection of distal tissues from a specific region of the fetal lungs helps to enrich the tip epithelial progenitor cells at the next step of this protocol.

**CAUTION**: Before beginning, it is mandatory to wear personal protective equipment (PPE) to maintain safety standards when working with freshly isolated human fetal-derived cells and sharp hazards in the cell/tissue culture clean bench.

1. Carefully cut edge regions of fetal lung tissues using fine forceps (11295-10, Fine Science Tools) and scissors (14060-09, Fine Science Tools) (Figure 2).
  a. Carefully cut up to 0.5 cm width × ∼1–3 cm length of the lung tissue away from the distal edges (Figures 2B–2D).
  b. Transfer it to a 1.5 mL microcentrifuge tube using forceps and chop into as small pieces, ∼1–2 mm in size, as possible inside the tube (Figure 2E).
2. Slightly cut the end tip of 1 mL sterile tip.
  a. Add 0.5 mL of tissue dissociation buffer into the tube and properly mix by gentle pipetting.
  b. Immediately transfer the fragmented tissues with the buffer inside the tip into 15 mL conical tubes containing 4.5 mL of the tissue dissociation buffer (Figure 2F).
3. Incubate for 40 min on a rocker with gentle swirling at 37°C. Gently triturate 2-30 times using 1 mL sterile tips every 20 min (Figures 2F and 2G).

**CRITICAL:** In contrast to non-epithelial cells, the epithelial cells forming distal tip structures are less likely to be dissociated into single cells. If required, increase the number of pipetting steps and length of incubation time for as full dissociation of the lung tissues as possible.

4. Ensure the tissues are mostly single-cell dissociated, under a bright field microscope. Then, add 10 mL of cold tissue washing buffer and mix well.
5. Centrifuge at 500 g for 5 min at 4°C in a swinging bucket rotor.
6. After removing the supernatant (see the pellet containing non-epithelial cells, e.g., red blood cells; Figure 2H), add 10 mL cold tissue wash buffer and carefully resuspend it.
7. Repeat the centrifugation (as Step 5).
8. Aspirate the supernatant and then, resuspend the pellet in 3 mL red blood cell lysis buffer.
9. Incubate at room temperature for 5 min.
10. Add 10 mL cold tissue wash buffer.
11. Filter the cell suspension through a 100 μm cell strainer.
12. Centrifuge at 500 g for 5 min at 4°C in a swinging bucket rotor.
13. Carefully remove the supernatant and resuspend the pellet (Figure 2I) with 10 mL tissue washing buffer.
14. Repeat the washing procedure from Step 12.
15. Resuspend the pellet with 300 μL cold MACS washing buffer.

***Note:*** For steps 15–26, follow the manufacturer’s instruction (CD326 (EPCAM) MicroBeads, human; 130-061-101; Miltenyi Biotec; https://www.miltenyibiotec.com/US-en/products/cd326-epcam-microbeads-human.html).

16. Add 100 μL FcR Blocking Reagent (130-059-901, Miltenyi Biotec) and incubate for 5 min at room temperature.
17. Add 100 μL CD326 (EpCAM) MicroBeads and mix carefully by gentle pipetting up and down.
18. Incubate for 30 min in the fridge at 4°C.
19. Wash the cells by adding 10 mL of MACS washing buffer and centrifuge at 300 g in a swinging bucket rotor for 10 min.
20. Aspirate the supernatant completely and resuspend the pellet in 500 μL MACS washing buffer.
21. Place LS column into a suitable MACS Separator.
22. Pre-rinse the LS column with 3 mL MACS washing buffer.
23. Apply the cell suspension onto the LS column. Discard flow-through containing unlabeled non-epithelial cells.
24. Wash the column by adding the 3 mL MACS washing buffer four times.

***Note:*** Repeat washing by adding MACS washing buffer as soon as the column reservoir is empty.

25. Remove the column from the separator and place it on a 15 mL conical tube.
26. Add 5 mL MACS washing buffer and immediately flush out the EPCAM+ cells. Collect the flow-through containing distal epithelial cells from the human fetal lungs.
27. Count the cell number collected using a hemocytometer.
28. Centrifuge at 500 g for 5 min at 4°C in a swinging bucket rotor.

***Note:*** Immediately move to step 29 on the same day.

### Primary *in vitro* culture of distal lung tip epithelial organoids

**Timing: 3 weeks**

This section describes the procedure for primary *in vitro* culture of the isolated distal lung tip epithelial cells.

29. Embed approximately 1 × 10^4^ of the isolated cells into a 20–25 μL single Matrigel droplet and immediately incubate the culture plate containing the Matrigel droplets in a 37°C incubator for 15 min. After confirming the droplets get fully solidified, add an appropriate volume of pre-warmed SN medium containing 10 μM Y-27632 (688000, Merck). Refresh fresh SN medium (without 10 μM Y-27632) every 2 days for 2 weeks until cells grow as 3D organoids.

**Table 1:** Recommended number of Matrigel droplets and volume of culture medium for different size culture vessels

	Culture vessel	No. of droplets	Volume of culture medium	Comment
Arrangement of the Matrigel droplets in a single well of multiwell plates, 12, 24, and 48-well plates	48-well plate	1	300 μL	Recommended for culturing single cells isolated by FACS
	24-well plate	3	500 μL	Recommended for regular subculturing
	12-well plate	7	1 mL	Recommended if mass production is required

***Note:*** The Matrigel needs to be thawed and kept on ice to reduce the viscosity and should be pipetted slowly to prevent bubble formation. The recommended number of Matrigel droplets and volume of the culture medium for different multiwell plates are in Table 1.

30. At 2 weeks of culture, remove the medium from the culture wells and add 1 mL of fresh basal medium. Using a pipette, gently break up the Matrigel containing the organoids until the organoids are released and the Matrigel fragments are no longer visible.

***Note:*** During the first 2 weeks of culture, a small number of single, non-epithelial cells may grow alongside the epithelial tip organoids in the Matrigel droplets. These single, non-epithelial cells are likely to remain undissociated from the Matrigel even after the Matrigel droplets have been disrupted. These cells can typically be removed during steps 30–34.

31. Collect the organoids along with the medium from each well. If necessary, combine the organoids from multiple wells into one 15 mL conical tube. Then, centrifuge at 500 g for 5 min at room temperature, in a swinging bucket rotor.
32. Carefully remove the supernatant along with the remaining Matrigel containing the dispersed non-epithelial cells.

***Note:*** Be careful not to suck out the whole epithelial organoid pellet from the bottom of the tube when removing the supernatant and the Matrigel. To ensure safe retention of the organoid pellets during suctioning, it is recommended to leave approximately 10% of the Matrigel volume at the bottom.

33. Add 200 μL fresh basal medium and gently pipette around 30 times using a 200 μL tip, to help remove the remaining Matrigel containing the non-epithelial cells and collect mainly the epithelial organoids. Then, add additional 1.8 mL fresh basal medium and centrifuge at 500 g for 5 min at room temperature.
34. Remove completely the supernatant and the remaining Matrigel, except the epithelial organoid pellet which will be present at the bottom of the supernatant.
35. Mix the organoid pellet with an appropriate amount of Matrigel and replate it in a ratio of 1:3–1:4; e.g., to split 1:3 the volume of Matrigel would be 60–75 μL, depending on the droplet size on the plate. Then, incubate the mixture in a 37°C incubator for at least 15 min, until the Matrigel becomes solidified.

***Note:*** Handling human fetal lung tissues requires attention to batch effects, which can affect cell viability and cellular heterogeneity. To successfully expand the epithelial tip organoids, we have empirically determined that using subculture ratios between 1:3 and 1:4 is the most effective.

**Figure 3:**
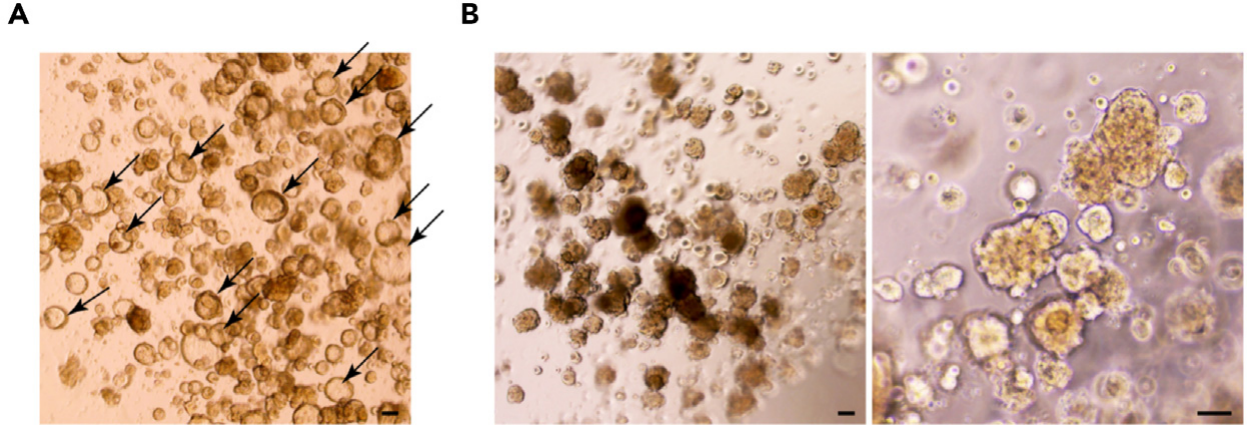
Enrichment of alveolar-fated, Lin^POS^ tip organoids (A) Bright-field images of the distal lung tip epithelial organoids at 3 weeks after the initial cell isolation, at the end of passage 1. (B) Lin^POS^ tip organoids enriched at 3 weeks after CD36+ sorting. The CD36+ cell fraction enriches to form the alveolar-fated, Lin^POS^ tip organoids at passage 2. Arrows, cystic, Lin^NEG^ tip organoids. Scale bars, 100 μm.

***Note:*** If required, extend the culture period longer than 1 week depending on size and density of the organoids until they grow enough in number, e.g. more than 1 million cells, to be ready for run FACS isolation.

36. Add fresh SN medium and refresh the medium every 2 days.
37. Culture the organoids for another 1 week (Figure 3A).

### Enrichment and expansion of Lin^POS^ tip progenitor organoids

**Timing: 3 weeks**

A mixture of distal lung tip epithelial organoids which are morphologically folded (lineage positive, Lin^POS^; alveolar lineage-fated, co-expressing the alveolar and lung progenitor markers) and cystic (lineage negative, Lin^NEG^) organoids which only express progenitor markers, will be observed at around 3 weeks of culture (Figure 1B, 1C, and 3A). This step describes how to successfully enrich the alveolar-fated Lin^POS^ tip organoids from the mixture and to remove the cystic Lin^NEG^ organoids (Figure 3B). To this end, CD36, a surface marker of late-stage lung tip epithelial progenitor cells will be used for flow cytometric cell (FACS)-sorting of the positive cells (Figures 1D and 1E).^1^

38. Remove the medium from the cultured wells and add fresh basal medium and resuspend them. Using a pipette, gently break up the Matrigel containing the organoids until the organoids are released and the Matrigel fragments are no longer visible.
39. Collect the organoids along with the medium from each well. If necessary, combine organoids from multiple wells into a new 15 mL conical tube. Centrifuge at 500 g for 5 min at room temperature.
40. Remove the supernatant and the residual amount of Matrigel which will be present at the bottom of the supernatant. Add 1 mL TrypLE to obtain a single cell suspension and resuspend organoids by gentle pipetting. Incubate at 37°C incubator for 5–10 min.
41. After incubation, gently pipette until the organoids are fully dissociated into single cells. Dilute with 9 mL fresh cold basal medium and centrifuge at 500 g for 5 min.

***Note:*** Ensure the cells become fully dissociated into single cells under the bright field microscope.

42. Remove the supernatant and resuspend the single cell pellet in an appropriate volume of cold FACS buffer.
43. Treat with CD36 antibody conjugated with a fluorophore, e.g., FITC (Mouse monoclonal anti-CD36, FITC conjugated; Thermo Fisher Scientific; Cat# 11-0369-42), in 1:100 ratio.
44. Sort CD36+ cells by FACS. Then, embed 1 × 10^4^ of the isolated CD36+ cells into a 25 μL single Matrigel droplet and add an appropriate volume of SN medium. Replace the SN medium every 2 days for 2 weeks until they grow as the folding, alveolar-fated Lin^POS^ tip organoids.

***Note:*** This step is important for ensuring the purity and fidelity of the organoid population. It is highly recommended to carefully manage the culture by manually removing any cystic organoids coming up from the culture at every passaging, even after the sorting. After 4–5 rounds of passaging, the Lin^POS^ tip organoid line will be further stabilized and cystic organoids will not appear. Troubleshooting 1 and 2.

45. **Expansion**: Passage the organoid line once a week, at a 1:3–1:4 ratio. For subsequent passaging, please follow the steps below:
  a. Collect the organoids along with the medium from wells. If necessary, combine the organoids from multiple wells into a new 15 mL conical tube. Then, centrifuge at 500 g for 5 min at room temperature.
  b. After removing the supernatant with any residual Matrigel, add 200 μL of fresh basal medium to the organoid pellet and pipette it up and down about 20∼30 times to break them up into smaller pieces.
    ***Note:*** Please ensure that the organoids are reduced in size, ranging from 50 to 200 μm when observed under the bright field microscope. If they are not adequately broken up, repeat the pipetting process an extra 20–30 times.
  c. Add 1.8 mL of fresh basal medium and centrifuge at 500 g for 5 min at room temperature.
  d. Remove the supernatant and the residual amount of Matrigel on the bottom. Mix the organoid pellet with an appropriate amount of Matrigel volume and embed them on a well plate - 20 to 25 μL per single Matrigel droplet.
    ***Note:*** The number of droplets can be determined by the initial number of the Matrigel droplets on the well(s) when collecting the organoids at step 45 (a). For example, if three droplets were initially used for the passaging, ∼9–12 droplets would be made at a 1:3–1:4 ratio. Please also see the Table 1 regarding the arrangement of the Matrigel droplets.
  e. Incubate the mixture in a 37°C incubator for at least 15 min, until the Matrigel becomes solidified.
  f. Add fresh SN medium and refresh the medium every 2 days. Passage the organoids once a week.

### Cryopreservation and thawing of Lin^POS^ tip progenitor organoids

**Timing: 15 min**

Cryopreservation of the cultured alveolar-fated Lin^POS^ tip progenitor organoids provides a practical advantage, which avoids the need to derive tip progenitor cells freshly from human fetal lung tissues. This step describes the procedure for the cryopreservation and subsequent thawing of alveolar-fated, Lin^POS^ tip progenitor organoids.

46. **Cryopreservation**: Once the cells have grown large enough – usually 4–5 days incubation after plating in Matrigel – remove the medium from the culture wells and add 1 mL of fresh basal medium. Using a pipette, gently break up the Matrigel containing the organoids until the organoids are released and the Matrigel fragments are almost invisible.
47. Collect the organoids along with the medium from the wells. If necessary, combine organoids from multiple wells into a new 15 mL conical tube. Centrifuge at 500 g for 5 min at room temperature.
48. After removing the supernatant with any residual Matrigel, add 200 μL of fresh basal medium to the organoid pellet and pipette it up and down about 20 times. Add 1.8 mL of fresh basal medium and centrifuge at 500 g for 5 min at room temperature.
49. Remove the supernatant and the residual amount of Matrigel on the bottom.
50. Resuspend the organoid pellet in 1 mL of Recovery Cell Culture Freezing Medium (1 mL per cryovial).
51. Place the vial(s) in proper cryostorage containers and gradually cool down in a −80°C freezer overnight and then transfer into liquid nitrogen for long-term storage.
52. **Thawing**: For thawing the cryopreserved organoids, quickly warm the vial(s) in 37°C water bath for 3–5 min until it becomes transparent, and then transfer into a 15 mL conical tube containing 9 mL of fresh basal medium.
53. Centrifuge at 500 g for 5 min at room temperature.
54. Mix the organoid pellet with an appropriate amount of Matrigel volume; 20–25 μL per single Matrigel droplet. Embed them on a well plate.
55. Incubate the mixture in a 37°C incubator for at least 15 min, until the Matrigel becomes solidified.

***Note:*** The recommended number of the Matrigel droplets at the step 54 can be determined by the number of Matrigel droplets collected when they were cryopreserved at the steps 50–51. If 3 droplets were used for cryopreservation, make 3 droplets when thawing. Please also see Table 1.

56. Add fresh SN medium and refresh the medium every 2 days.
57. After 2 weeks, subculture the organoid lines.

### Lentivirus transduction of SFTPC-GFP reporter into Lin^POS^ tip progenitor organoids

**Timing: 10 days**

The presence of the alveolar type 2 lineage marker, SFTPC, is a hallmark of late-stage tip epithelial cells after 16 pcw in human fetal lungs. Thus, the introduction of a fluorescence reporter, such as GFP, under the control of the *SFTPC* promoter offers numerous advantages for exploring signaling pathways, cell-cell interactions, cell fate determination, disease modeling, and more.^1^ In this context, we present an *optional* method involving lentivirus transduction to incorporate the *SFTPC*-GFP reporter into the Lin^POS^ tip progenitor organoids.

58. **Lentiviral transduction (*optional*)**: Remove the culture medium from six to eight Matrigel droplets containing the organoids and resuspend them in fresh basal medium using a pipette by gently breaking up until the organoids are released and the Matrigel fragments are no longer visible.
59. Collect the organoids along with the medium and combine them into a new 15 mL conical tube. Centrifuge at 500 g for 5 min at room temperature.
60. Remove the supernatant and the residual amount of Matrigel which will be present at the bottom of the supernatant. Add 1 mL TrypLE and resuspend organoids by gentle pipetting. Incubate at 37°C incubator for 5–10 min.
61. Dilute with 9 mL fresh cold basal medium and centrifuge at 500 g for 5 min.
62. Remove the supernatant and resuspend the single cell pellet in 1 mL of the SN medium containing 10 μM Y-27632.
63. Add lentivirus particles harboring SFTPC-GFP reporter (Addgene #36450 or Addgene #201681) at an MOI (multiplicity of infection) of 1, to the medium containing the cells and mix properly by gentle pipetting.
64. Transfer the mixture of the cells and the virus in the SN medium to one or two well(s) of a 24 well-plate using 1 mL tip.

**CRITICAL:** No Matrigel droplet is required because the Matrigel disturbs lentiviral infection of the cells.

65. Incubate at 37°C humidified incubator for 48 h without the Matrigel.

***Note:*** Ensure some cells express the GFP signal at 48 h after infection under a fluorescence microscope. If no GFP was observed, prepare the virus again.

***Note:*** After 48 h after the infection, the cells will form a tiny aggregate.

66. Collect all the infected cells along with the medium and transfer them into a new 15 mL conical tube.
67. Centrifuge at 500 g for 5 min at room temperature. Aspirate and fully remove the supernatant.
68. Add 1 mL TrypLE and resuspend the aggregates by gentle pipetting. Incubate at 37°C incubator for 5–10 min. After incubation, gently pipette until the aggregates are fully dissociated into single cells.

***Note:*** Ensure the cell aggregates become fully dissociated into single cells under the bright field microscope.

69. Dilute with 9 mL fresh cold basal medium and centrifuge at 500 g for 5 min.
70. Remove the supernatant and mix the cells with 120∼200 μL of Matrigel - 20 to 25 μL per single Matrigel droplet. Then, immediately embed them on a well plate.
71. Culture the infected cells in fresh SN medium containing 10 μM Y-27632 for the first 2 days.
72. Refresh the SN medium without 10 μM Y-27632 for another 4–6 days.
73. **Purification of SFTPC-GFP+ cells**: At 8–10 days after the infection, remove the culture medium from the wells and add 1 mL fresh basal medium. Then, resuspend them using a pipette by gently break up the Matrigel containing the organoids until the organoids are released and the Matrigel fragments are no longer visible.
74. Collect the organoids along with the medium from the wells and combine them into a new 15 mL conical tube. Centrifuge at 500 g for 5 min at room temperature.
75. Remove the supernatant and the residual amount of Matrigel which will be present at the bottom of the supernatant.
76. Add 1 mL TrypLE and resuspend organoids by gentle pipetting and then incubate in 37°C incubator for 5–10 min. After incubation, gently pipette until the organoids are fully dissociated into single cells.

***Note:*** Ensure the cells become fully dissociated into single cells under a bright field microscope.

77. Dilute with 9 mL fresh cold basal medium and centrifuge at 500 g for 5 min.
78. Remove the supernatant and resuspend the single cell pellet in 200 μL cold FACS buffer.

***Note:*** Prepare non-infected, non-fluorescent cells separately as a negative control.

79. Sort GFP positive cells.
80. Embed 1 × 10^4^ of the isolated GFP^+^ cells into a 25 μL single Matrigel droplet and add an appropriate volume of SN medium containing 10 μM Y-27632.
81. Refresh fresh SN medium without 10 μM Y-27632 every 2 days. Expand the SFTPC-GFP^+^ Lin^POS^ tip progenitor organoids as required for further analysis.

***Note:*** In case any cystic organoids appear on a dish after sorting manually remove them by picking out.

### AT2 fate differentiation of Lin^POS^ tip progenitor organoids

**Timing: 7 days**

82. Prior to inducing alveolar type 2 cell fate differentiation, passage the cultured Lin^POS^ tip organoids to make the size of the organoids not too big; smaller than 100 μm if possible.
83. Add fresh SN medium and culture for 1–2 days in the SN medium (Figure 4A).

**Figure 4:**
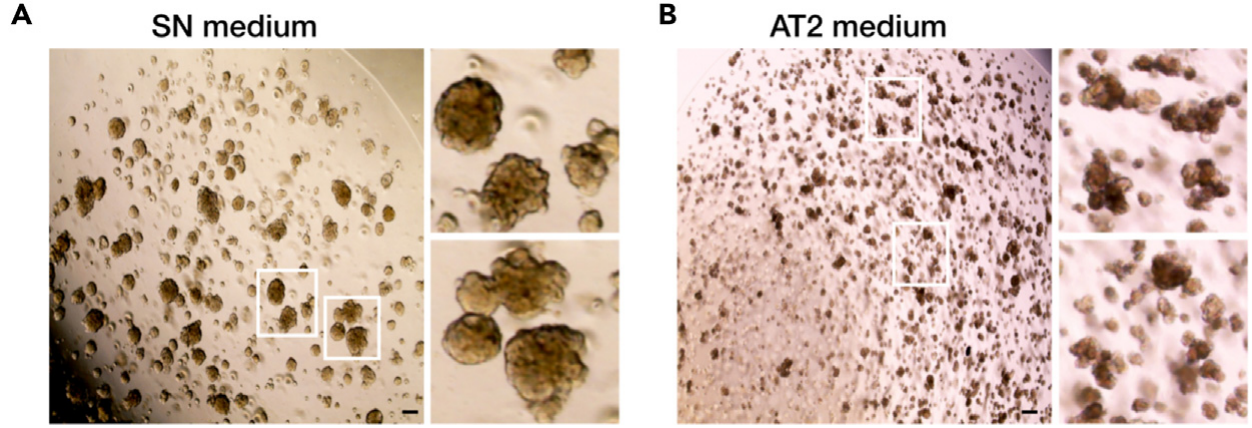
AT2 cell fate differentiation of Lin^POS^ tip progenitor organoids By switching medium conditions from SN medium (A) to AT2 medium (B) the Lin^POS^ tip organoids readily differentiate to an AT2 cell lineage within 1 week. See also Figure 5 and Lim et al. 2023. Scale bars, 100 μm.
84. Switch the SN medium to AT2 medium and culture for 1 week without passaging (Figures 4B and 5A).

**Figure 5:**
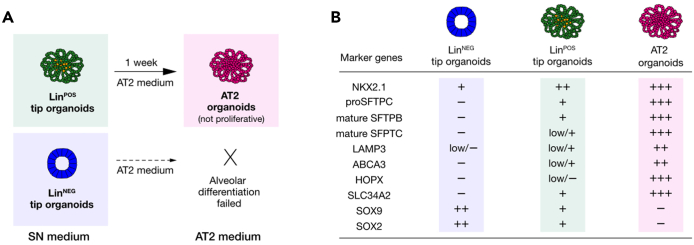
Comparison of Lin^POS^ and Lin^NEG^ tip progenitor organoids and AT2 organoids (A) The Lin^POS^ tip organoids can differentiate to an AT2 cell lineage by switching the culture medium conditions from the SN to AT2 medium, whereas the Lin^NEG^ tip organoids fail AT2 fate differentiation. See also Figure 4 and Lim et al. 2023. (B) A list of marker genes/proteins that are differentially expressed in each organoid line. Alveolar lineage markers include NKX2.1, pro SFTPC, mature SFTPB, mature SFTPC, LAMP3, ABCA3, HOPX, and SLC34A2. Tip progenitor markers include SOX9 and SOX2.
85. Evaluate the AT2 differentiation by qRT-PCR and immunostaining/western blot (Figures 1F and 1G).

## Expected outcomes

Using this protocol, the distal lung tip progenitor cells from late stage of human fetal lungs, 19–21 pcw, can be successfully cultured *in vitro* and expanded under the defined culture condition. During the first 3 weeks, the isolated tip epithelial progenitor cells give rise to a mixture of organoids, lineage positive (Lin^POS^) and negative (Lin^NEG^), which both commonly express tip progenitor markers, SOX9 and SOX2 as *in vivo* tip epithelium shows (Figure 5B)^1^^,^^2^^,^^7^; As reported, the Lin^POS^ tip organoids are comprised of largely alveolar lineage fated progenitor cells (Figure 1; ^1^). The Lin^NEG^ tip organoids do not show any lineage marker expression but only express SOX9 and SOX2 (Figure 1). CD36, a surface marker of alveolar fated tip epithelium in the late-stage lung tissues, can be used for enriching the Lin^POS^ tip organoids and removing the Lin^NEG^ cells from the culture (Figure 3). Under the alveolar type 2 (AT2) medium, the Lin^POS^ tip organoids successfully differentiate into the AT2 organoids that can produce and secrete the mature form of surfactant proteins and express a high level of surfactant protein synthesis-associated genes, such as *LAMP3* and *ABCA3* (Figures 4 and 5B)^1^^,^^8^; By contrast, the Lin^NEG^ organoids fail to differentiate in this AT2 differentiation protocol. Since the Lin^POS^ tip organoids can be expanded and can differentiate into AT2 cell lineages, researchers can generate a large number of AT2-like cells for next-generation sequencing, gene editing, or protein assays, which typically require a large input of cells to investigate alveolar differentiation mechanisms at the molecular level.

## Limitations

We have frequently observed batch-to-batch variation, even when using fresh lung tissues of the same developmental age. This may be due to the genetic variation between samples. Or due to the tip progenitor cells being obtained from human fetal lungs that may have different tissue conditions upon arrival at the lab, such as fragmentation and/or hemorrhage, which could affect the viability of isolated cells. In addition, the estimated developmental age of the fetus may not always be accurate, which can affect the success of late-stage tip organoid line establishment. We recommend using tissues from later developmental stages, if possible, at least 19 pcw or older.

## Troubleshooting

### Problem 1

**Figure 6:**
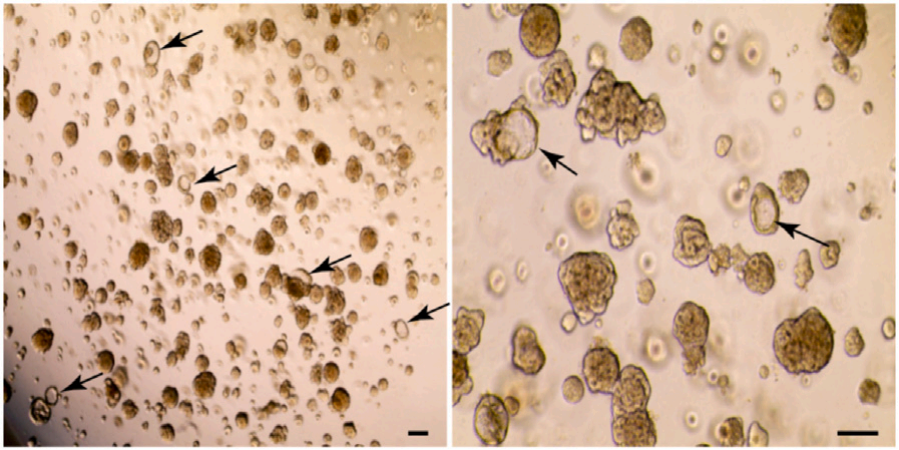
Repeated emergence of Lin^NEG^ cystic organoids Bright-field images showing repeated emergence of Lin^NEG^ cystic organoids (arrows) after enrichment of the CD36+ cells. Scale bars, 100 μm.

***Repeated emergence of Lin*^*NEG*^*cystic organoids***. The purification step of CD36^+^ cells from the cultured organoids is essential for enriching the alveolar-fated tip progenitor population. However, depending on batch-to-batch variation, lineage-negative, cystic organoids may still be observed during multiple rounds of organoid passaging after CD36^+^ cell purification by FACS. As seen in Lim et al. (2023), the SFTPC-GFP expressing tip cells can grow into Lin^NEG^ tip organoids in the SN medium, although this was observed infrequently (Figure 6).

We consider that this is a spontaneous process likely due to the current medium composition containing abundant amount of FGFs and EGF. We strongly recommend the suggested potential solution below, as the cystic organoids grow relatively faster than the alveolar-fated tip progenitor organoids.

### Potential solution

• Manually remove the cystic organoids from the culture by picking them out using a fine tip of pipette at every passaging under a dissection microscope.
• Repeat the purification step of CD36+ cells by FACS.

### Problem 2

**Figure 7:**
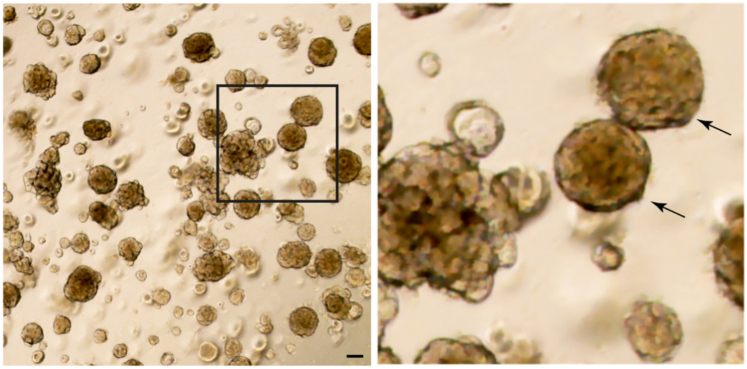
Airway fated organoids Bright field images showing non-alveolar cell lineage organoids, relatively dark and spheroid in shape (arrows). Scale bars, 100 μm.

***Observation of airway fated cells within the Lin*^*POS*^*organoids***. The observation of TP63 positive, airway fated cells in the Lin^POS^ organoids is likely due to the self-organizing characteristics of the late-stage tip progenitor cells, which can generate non-alveolar cell lineages, including airway fates, during the prolonged culture when a regional Wnt-low condition is temporarily formed within the organoids.^1^ The Lin^POS^ organoids that overgrow to a large size, ranging from 100 to 400 μm in a spheroid shape, probably have more TP63+ cells (Figure 7). Therefore, it is highly recommended that the size of the organoids should be regularly monitored and managed by subculturing once a week to prevent activation of the airway fate program.

### Potential solution

Regularly monitor the size of the organoids and subculture the organoid line once a week at least. If required, pick the relatively dark and spheroid shaped organoids out from the culture using fine tips. If this is a persistent problem passage organoids via dissociation to single cells using TrypLE, rather than by breaking into small pieces, to limit organoid size.

## Resource availability

### Lead contact

Further information and requests for resources and reagents should be directed to and will be fulfilled by the lead contact, Emma L. Rawlins (erl21@cam.ac.uk).

### Technical contact

Further technical information for following the detailed steps should be directed to the technical contact, Kyungtae Lim (ktlim492@korea.ac.kr).

### Materials availability

Lung organoid lines used in this study are available from the lead contact, Emma L. Rawlins (erl21@cam.ac.uk), with a completed Materials Transfer Agreement.

### Data and code availability

Sequencing data have been deposited at ArrayExpress and GEO and are publicly available. Accession numbers are listed in the key resources table. Processed single cell sequencing data reported in this paper are available at https://fetal-lung.cellgeni.sanger.ac.uk/.
